# The potential of decision support systems to improve risk assessment for pollen beetle management in winter oilseed rape

**DOI:** 10.1002/ps.4069

**Published:** 2015-08-26

**Authors:** Andrew W Ferguson, Matthew P Skellern, Andreas Johnen, Julia‐Sophie von Richthofen, Nigel P Watts, Eileen Bardsley, Darren A Murray, Samantha M Cook

**Affiliations:** ^1^ AgroEcology DepartmentRothamsted ResearchHarpendenHertfordshireUK; ^2^ proPlant GmbHMünsterGermany; ^3^ Formerly at Bayer CropScience LtdCambridgeUK (now retired); ^4^ VSN International LtdHemel HempsteadHertfordshireUK

**Keywords:** Brassica napus, Brassicogethes aeneus, decision support systems, integrated pest management, Meligethes aeneus, monitoring, phenological model

## Abstract

**BACKGROUND:**

The reliance on and extensive use of pyrethroid insecticides have led to pyrethroid resistance in pollen beetle (Meligethes aeneus). Widespread adoption of best practice in pollen beetle management is therefore needed. Decision support systems (DSSs) that identify the risk period(s) for pest migration can help to target monitoring and control efforts, but they must be accurate and labour efficient to gain the support of growers. Weather data and the phenology of pollen beetles in 44 winter oilseed rape crops across England over 4 years were used to compare the performance of two risk management tools: the DSS proPlant expert, which predicts migration risk according to a phenological model and local weather data, and ‘rule‐based advice’, which depends on crop growth stage and a temperature threshold.

**RESULTS:**

Both risk management tools were effective in prompting monitoring that would detect breaches of various control thresholds. However, the DSS more accurately predicted migration start and advised significantly fewer days of migration risk, consultation days and monitoring than did rule‐based advice.

**CONCLUSION:**

The proPlant expert DSS reliably models pollen beetle phenology. Use of such a DSS can focus monitoring effort to when it is most needed, facilitate the practical use of thresholds and help to prevent unnecessary insecticide applications and the development of insecticide resistance. © 2015 Rothamsted Research Ltd. *Pest Management Science* published by John Wiley & Sons Ltd on behalf of Society of Chemical Industry.

## 1 INTRODUCTION

Pesticide stewardship in the management of pollen beetles (*Meligethes aeneus* F.) in oilseed rape (OSR) (*Brassica napus* L.) has become an increasingly urgent issue in light of the threat posed by insecticide resistance.^1^, ^2^, ^3^, ^4^ This risk and its potential consequences for farm incomes are accentuated in Europe by increased production of the crop; total European Union production of rapeseed increased from 18.9 million t in 2008 to 21.0 million t in 2013 (Eurostat, http://epp.eurostat.ec.europa.eu).

Good decision support is an important element of integrated pest management strategies, and the adoption of these strategies, where available, is encouraged in Europe as part of the EU Directive to promote the sustainable use of pesticides (2009/128/EC; effective since 1 January 2014). Decision support systems enable growers and advisors to determine levels of risk to the crop, monitor appropriately to assess pest abundance and take action if necessary, based on thresholds. Here we seek evidence that use of a web‐based DSS system can improve risk assessment for pollen beetle management in winter oilseed rape (WOSR).

Pollen beetles cause damage to flower buds by feeding in them, resulting in bud abscission and loss of yield.^5^, ^6^ They are the major target of spring‐applied insecticides,^7^ yet data exist to suggest that insecticide treatment for pollen beetles is in marked excess of the frequency with which their populations reach control threshold levels. According to a UK survey in 2008 by CropMonitor^TM^ (http://www.cropmonitor.co.uk/wosr/surveys/wosr‐sprPest_gyb08.cfm), the lower control threshold of 5 beetles plant^−1^ was breached at only one of 48 WOSR fields sampled across England and Wales, and the standard 15 beetles plant^−1^ threshold was never attained at these sites. However, in the same year, an average of 0.3 insecticide applications per field were targeted against pollen beetles in oilseed rape in Great Britain.^8^ It appears that many growers may apply prophylactic ‘insurance’ sprays, rather than following advice for monitoring and treating according to thresholds. This is probably largely due to the extended period over which monitoring is required (immigration can occur over a period of about 3 weeks) and the perception that the recommended monitoring procedure is laborious. According to the rule‐based advice current in the United Kingdom at the time of this study, growers were recommended to monitor the number of pollen beetles per main shoot at the headland and midfield during the bud stage, when crops are susceptible to damage.^9^, ^10^ It was advised that pollen beetles fly at air temperatures of 15 °C or above and migrate to crops in March and April, and this was the only guidance by which to focus crop monitoring.

Not only is rule‐based advice that relies on a simple temperature threshold likely to lead to an excessive requirement for monitoring, it is also likely to take insufficient account of the significant factors governing the timing of migration. The 15 °C flight temperature threshold advised in the United Kingdom^9^, ^11^ accords with the generally accepted threshold for mass migration of pollen beetles to winter oilseed rape crops in Europe (e.g. Fritzsche^12^), but significant migration has been reported at lower air temperatures.^13^ Šedivy and Kocourek^14^ reported that mass flight could occur at temperatures above 13.5 °C. Ferguson *et al.* 15, 16 found evidence for pollen beetle flight at 12 °C within a field plot of WOSR and at 10.9 °C in a laboratory bioassay. This suggests that other factors such as wind speed, insolation or precipitation may interact with air temperature to influence the microclimate experienced by pollen beetles in field conditions.^17^

Decision support systems (DSSs) for pest management are computer‐based information systems that assist the grower in decision‐making in crop protection. They may incorporate models of pest phenology and/or damage risk, are parameterised by multiple variables, and they generate risk assessments and other information upon which the grower can act. When implemented effectively, they can improve crop protection and minimise the input and cost of pesticides.^18^ A DSS that accurately identifies the period of risk by modelling the dynamics of the population of pollen beetles local to the crop could allow monitoring to be more accurately timed and therefore less onerous. This could increase the use of thresholds and lead to reductions in unnecessary insecticide treatments and in selection for insecticide resistance.^19^

‘proPlant expert’ (http://www.proplantexpert.com) is a web‐based DSS developed in Germany that alerts the user to the start of pest migration and its progress. It provides local 3 day forecasts of pest migration risk and indicates days when crop monitoring is needed. proPlant expert's forecasts are based on phenological models developed from historical data on pest abundance in crops and a sophisticated use of weather variables.^17^, ^20^ The model for pollen beetles is driven by daily records of air temperature, rainfall, sunshine and wind speed, automatically downloaded from local meteorological stations. proPlant expert is widely used commercially for WOSR in Germany, Austria, Belarus, the Czech Republic, France, Poland and Sweden, and output from its pollen beetle model has recently been made available in the United Kingdom at http://www.bayercropscience.co.uk/ (see also Ferguson and Cook^21^). It is currently the only commercially available phenological‐model‐based DSS for pollen beetles in Europe.

Here we report on a 4 year study to compare the performance of simple rule‐based advice and a phenological‐model‐based DSS in relation to pollen beetle management, taking as a model system the current rule‐based recommendations in the United Kingdom and the proPlant expert DSS. Weather data and the phenology of pollen beetles on sticky traps and in winter OSR crops across central and eastern England were used to compare the accuracy with which these two risk management tools identified migration risk, the intensiveness of consultation required and the crop monitoring effort that each advised. We use three notional control threshold levels of pollen beetle numbers as benchmarks (two, five and 15 beetles per main raceme), reflecting the range of control thresholds currently recommended in different European countries.^6^, ^22^

## 2 EXPERIMENTAL METHODS

### 2.1 Field observations

The phenology of the beetles on winter oilseed rape crops in spring was assessed on a total of 169 commercially managed fields in the United Kingdom during March to early May in 2008–2011, as part of a project to develop an integrated pest management strategy for the control of pollen beetles.^23^ Pollen beetles were regularly monitored at each field by counting on plants and/or yellow sticky traps. Beetles on plants were counted on ten main racemes equally spaced along each of two 30 m transects into the field at the upwind and downwind edges of the crop (relative to the prevailing west‐south‐westerly wind). Yellow sticky traps were placed 3 m into the crop at the upwind and downwind sides of the field, facing out of the crop. The growth stage of the crop was recorded at each assessment according to the BBCH scale of Lancashire *et al.* 24 Much of the monitoring programme was undertaken by volunteer farmers and advisers, and the intensity of monitoring at each site was determined by the commitment they were able to make.

For this study, a subset of 44 fields in eastern and central England were chosen (two, ten, 12 and 20 fields in 2008, 2009, 2010 and 2011, respectively) (supporting information Fig. S1). These fields were selected because pollen beetles were monitored both on plants and on sticky traps approximately twice weekly during the green‐to‐yellow bud stage (BBCH growth stages 51 to 59), the period during which WOSR is susceptible to pollen beetle damage.^5^, ^11^ For each field site, the mean number of pollen beetles per plant was calculated for each monitoring date and compared with the two spray thresholds that were advised in the United Kingdom until 2011 (5 and 15 beetles plant^−1^).^9^, ^10^ An additional threshold of 2 beetles plant^−1^ was included to reflect the lower range of thresholds in current use in European countries.^6^, ^22^

The frequency of monitoring requested was minimised (to twice weekly) in the interests of encouraging wide participation of volunteers, and it was therefore necessary to estimate some phenological parameters. The period when plants in each field were at the bud development growth stages (51 to 59), delimiting the period when plants are at risk of pollen beetle damage, was estimated by interpolation from growth stage data recorded on the twice weekly monitoring dates, taking into account the progression of growth stages at other sites in the same year. It was assumed that any breach of a pollen beetle threshold took place on the monitoring date on which it was observed. This conservative assumption is independent both of the risk management tool used (rule‐based advice or DSS) and of the weather data on which they were based. Any consequent delay in the recognition of a threshold breach is likely to affect the performance assessment of each management tool equally.

### 2.2 Weather data

Weather data were obtained from the UK Met Office or from farmer‐operated meteorological stations within 1–80 km (average 16 km) of each sampled field. The proPlant phenological model requires daily measurements of minimum and maximum air temperature (°C), average air temperature (°C), rainfall (mm), sunshine (h) and average wind speed (m s^−1^).

### 2.3 Risk management tools

#### 2.3.1 proPlant expert DSS

**Figure 1:**
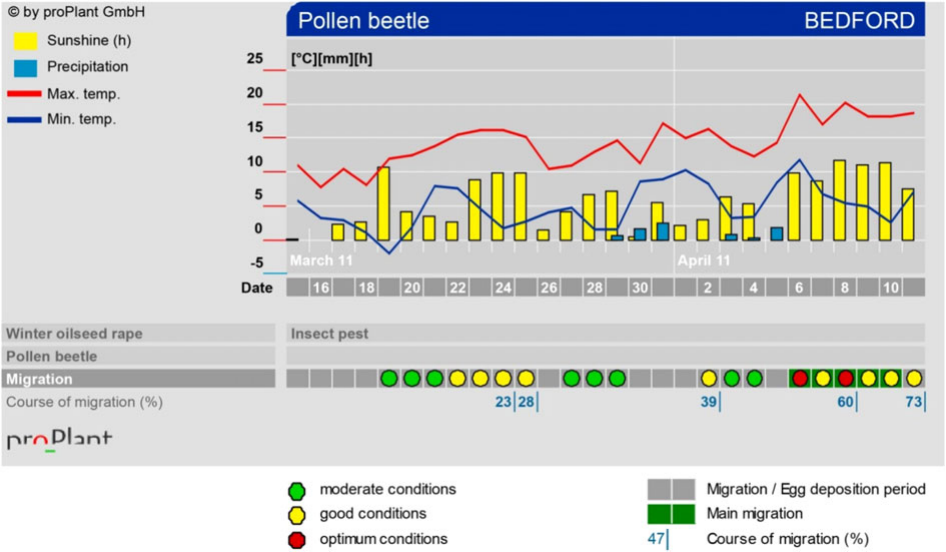
Example of proPlant expert's output for pollen beetle migration using meteorological data from the Bedford (UK) weather station in 2011. The bar and line graphs at the top illustrate weather data. The output of the migration model is given below the weather data on the migration bar, which carries information on the likelihood of pollen beetle migration each day. The background colour of the bar indicates whether the day falls within the migration period for the insect: pale grey indicates that it does not, and dark grey that it does (as seen throughout the period illustrated here). The degree of risk of pollen beetle migration into winter oilseed rape crops is indicated on the migration bar using a traffic‐light system of green, yellow and red dots for each day, signifying moderate, good or optimal migration conditions, respectively. Days when it is recommended that the crop should be monitored are indicated by a vertical blue line beneath the migration bar. The line is accompanied with an estimate of the percentage of the pollen beetle population that has migrated from overwintering sites to date. When viewed in greyscale, green spots on the migration bar appear mid‐grey, yellow spots are pale grey and red spots are dark grey.

The standard version of the proPlant model marketed in Europe in 2011 was used throughout this study. proPlant expert's web‐based user interface provides a graphical display of its model output, showing forecasts of pollen beetle migration risk and weather data for the day the system is consulted and for the following 2 days (Fig. 1). proPlant expert is updated daily with forecast and recorded weather data, and the model's output for the previous month is shown in the same graphic as the forecast. The ‘migration bar’ carries information on whether each day falls within the migration period for the insect and indicates the degree of risk of pollen beetle migration into WOSR crops using a traffic‐light system of green, yellow and red dots, signifying moderate, good or optimal migration conditions, respectively (Fig. 1). For this study, days when it is recommended that the crop should be monitored are indicated by a vertical blue line beneath the migration bar. The line is accompanied with an estimate of the percentage of the pollen beetle population that has migrated from overwintering sites to date. This information is intended to allow the user to estimate the potential magnitude of any further migration, relative to the number of beetles already in the crop, and is provided via another menu in some commercially available proPlant expert versions. proPlant expert advises that monitoring is necessary only on days with good or optimal migration conditions, and that monitoring should start on the day with the first yellow or red dot. Thereafter, if a contiguous series of days with good or optimal migration conditions occurs, monitoring is needed every third day and on the last day in the series.

#### 2.3.2 Rule‐based advice

The rule‐based advice against which the proPlant expert DSS was compared was that available to UK growers during the period of the study, i.e. that WOSR crops are at risk at bud stage, before flowers are open, and that pollen beetles start to fly at 15 °C.^9^, ^10^

### 2.4 Assessment and comparison of risk management tools

The performance of the two risk management tools was compared for each of the three selected control thresholds (2, 5 and 15 beetles plant^−1^) separately. For each site and for each risk management tool, the dataset was delimited by the period between growth stage 51 (or the start of field monitoring if later) and the day any breach of threshold would have been detected had the advice of the management tool been followed. If no threshold breach was detected, the whole period from growth stages 51 to 59 was included. Observations following any spring insecticide applications were excluded from the analysis.

Most assessments and comparisons of the two management tools were made a posteriori, using known pollen beetle phenology and known weather data. This enabled selection of the subset of sampled sites with sufficient pollen beetles for analysis and indicated the locations for which weather records were needed. An analysis of the performance of the two management tools in real time was also made. Each day from 2 March to 21 April 2011, 3 day forecasts of weather parameters were obtained for the UK Met Office Bedford site. These parameters were used to provide 3 day proPlant expert forecasts of pollen beetle migration risk and to indicate the likelihood of exceeding the 15 °C migration threshold used in the rule‐based advice. Using these data, the real‐time performance of each risk management tool in relation to field monitoring data from nine sites within 50 km of Bedford was assessed and compared over 41 days from 10 March to 20 April 2011, the period estimated by proPlant expert to encompass all pollen beetle migration at Bedford. This approach takes into account the uncertainty inherent in weather forecasts, as well as the accuracy of the risk model. It was used both to assess the accuracy of risk predictions and to validate the a posteriori approach applied to the main dataset.

#### 2.4.1 Performance criteria for comparisons between risk management tools

Several performance measures were compared for ‘rule‐based advice’ and for the proPlant expert DSS:

- Effectiveness in prompting monitoring that would have led to detection of breached control thresholds.
- Timeliness in prompting the detection of these threshold breaches.
- Ability to warn of the start of migration. This was assessed by comparing the proportion of sites where migration risk was advised prior to or coincident with the first beetles in field samples.
- Accuracy of predicted migration risk in comparison with assessments of risk made using the risk management tool a posteriori, using known weather data.
- Number of days when significant migration risk, forecast consultation or crop monitoring were advised.
  - Migration risk. The number of days of significant pollen beetle migration risk that were advised after the start of growth stage 51 and prior to each threshold breach (or until the end of growth stage 59) was counted. Days with significant migration risk were taken to be all those with a maximum temperature of ≥15 °C for rule‐based advice and all those with yellow or red dots for the proPlant expert DSS.
  - Forecast consultation. The number of days when a weather forecast consultation or a DSS forecast consultation was advised during growth stages 51 to 59, up to the dates that any threshold breaches would be detected, was counted. For rule‐based advice, all days during the susceptible plant growth stage (51 to 59) were taken to be ‘forecast consultation days’, i.e. days when the weather forecast should be consulted to determine whether the temperature was likely to reach 15 °C. DSS forecast consultation days were designated according to the following proPlant guidance: the DSS should first be consulted on the day that crop growth stage reaches 51; thereafter, it should be consulted either every third day or on any day when a coloured dot on the migration bar has been indicated by a previous consultation, whichever is more frequent; the final proPlant expert consultation should be made on the day after migration is predicted to be complete or consultation should cease at flowering, whichever is earlier.
  - Crop monitoring. The number of crop monitoring days advised during growth stages 51 to 59, up to the dates that any threshold breaches would be detected, was counted. For rule‐based advice, all days with a maximum temperature of ≥15 °C during growth stages 51 to 59 were taken to be monitoring days. The DSS proPlant expert indicates specific monitoring days on its user interface (see Section 2.3.1 above).

#### 2.4.2 Statistical analysis

The number of days of migration risk advised, the number of consultation days advised and the number of monitoring days advised prior to each threshold breach were analysed using bivariate mixed models, accounting for variation between sites and years. For the comparison of the real‐time performance of rule‐based advice and the DSS, analysis of variance was used. Both analyses were done using GenStat, v.14 (VSN International, 2011).

## 3 RESULTS

### 3.1 The dataset

The mean duration of the comparison of risk management tools at the 44 sites included in the study was 17 days, and the mean interval between field samples was 3.7 days (Table 1). The mean distance between the sampling site and the nearest weather station was 16 km, with a mode of 1 km and a range of 1–80 km (Table 1). Although the 15 beetles plant^−1^ threshold was breached at only one site, the 5 and 2 beetles plant^−1^ thresholds were breached at 19 and 36 sites (43 and 82%), respectively; sufficient to provide a good test of the performance of each management tool.

**Table 1:** Summary of sampling site parameters

Year	Number of sites	Mean kilometres to weather station (range)	Mean days duration of risk management tool comparison (range)	Mean days sample interval (range)
2008	2	9 (1–17)	22 (21–23)	3.8 (3–6)
2009	10	1 (0)^a^	19 (8–33)	3.7 (1–7)
2010	12	15 (1–48)	21 (10–31)	3.8 (2–7)
2011	20	26 (1–80)	13 (5–19)	3.7 (2–7)
All years	44	16 (1–80)	17 (5–33)	3.7 (1–7)

a All sites were recorded to be either 1 km or <2 km from the sampling site, so there is no range.

### 3.2 Relative performance of proPlant expert DSS and rule‐based advice

#### 3.2.1 Effectiveness in prompting monitoring that would have led to detection of breached control thresholds

The performance of both rule‐based advice and the DSS in prompting monitoring that would recognise breaches of thresholds was very good. All breaches except for two at the 2 beetles plant^−1^ threshold in 2010 would have been recognised using either risk management tool. Even these two failures were probably an artefact arising from the experimental monitoring regime. At Wicken, experimental monitoring detected that the threshold had been breached on 12 April, a day when the maximum temperature did not reach 15 °C and when neither management tool recommended monitoring; no further monitoring was recommended before flowering. Had monitoring occurred on 10 April, in accordance with either management tool, the threshold breach would almost certainly have been detected then, as it was the last of three days with temperatures above 15 °C. Similarly, at Broadmead field, Woburn, proPlant expert advised monitoring on 28 April, a day with optimum migration conditions. However, experimental monitoring indicated that the threshold breach did not occur until 2 days later, by which time proPlant expert had advised that migration had ceased and monitoring was no longer necessary. Responsive monitoring according to the recommendations of either management tool would almost certainly have prompted recognition of the breached threshold.

#### 3.2.2 Relative timeliness of threshold breach detection

The detection of breached thresholds would have been slightly delayed using the DSS as opposed to rule‐based advice (2 beetles plant^−1^ threshold: mean delay 0.68 days, SE 0.145, n = 34; 5 beetles plant^−1^ threshold: mean delay 0.84 days, SE 0.158, n = 19; 15 beetles plant^−1^ threshold: 2 days delay, n = 1).

#### 3.2.3 Ability to warn of the start of migration

The first records of pollen beetles on sticky traps or on plants in experimental fields were consistently preceded by or accompanied with a proPlant expert coloured‐dot risk warning (Table 2). Sticky traps caught beetles before they were found on plant samples at 91% of sites and were a better indicator of the start of migration than was the first day with a temperature of ≥15 °C. The latter performed similarly to proPlant expert's higher‐level yellow or red risk warnings of good or optimum migration conditions (Table 2). The attainment of green bud stage, the growth stage when the crop becomes susceptible to pollen beetle damage, was not a good indicator of the start of pollen beetle migration (Table 2).

**Table 2:** Ability of different indicators to warn of the start of pollen beetle migration

Pollen beetle sample type	Percentage of sites where different indicators preceded or coincided with the first pollen beetles in samples				
	First beetles found on sticky traps	Date average crop growth stage reached green bud	First occasion with temperature of ≥15 °C	First proPlant dot of any colour	First yellow or red proPlant dot
First beetles found on plants	89.5	38.6	68.2	100.0	65.9
First beetles found on sticky traps	—	22.7	63.6	100.0	52.3
Number of sites sampled	19^a^	44	44	44	44

a Sites are included in this comparison only if no pollen beetles were detected on sticky traps or on plants at the start of sampling.

#### 3.2.4 Accuracy of predicted migration risk in comparison with assessments of risk made with the risk management tool a posteriori, using known weather data

For each risk management tool, 3 day forecasts of good migration conditions showed a high degree of accuracy compared with a posteriori assessments using the same tool (Table 3). Their accuracy declined only moderately with prediction further into the future. Over 92% of weather forecasts correctly predicted whether air temperature would exceed 15 °C on the day that the forecast was issued, declining to 85% for the forecast for 2 days ahead (Table 3). Levels of accuracy of proPlant expert forecasts of good migration conditions (yellow or red dot) were similar to the accuracy of temperature forecasts, consistent with its model's use of forecast weather data. There was no overall tendency either to underestimate or to overestimate the likelihood of good migration conditions, but both management tools tended to underestimate the risk 2 days ahead (Table 3).

**Table 3:** Accuracy of prediction of good pollen beetle migration conditions relative to a posteriori assessment of migration conditions

	Rule‐based advice: forecast of maximum temperature of ≥15 °C			proPlant DSS: forecast of good migration conditions (yellow or red dot)		
	Day 0	Day +1	Day +2	Day 0	Day +1	Day +2
% Predictions accurate	92.7	90.0	84.6	92.7	92.5	87.2
% Predictions overestimated^a^	7.3	7.5	5.1	2.4	2.5	0.0
% Predictions underestimated^a^	0.0	2.5	10.3	4.9	5.0	12.8
n	41	40	39	41	40	39

a Overestimated: the prediction forecast good migration conditions that did not occur when assessed a posteriori; underestimated: failure to predict good migration conditions that did occur.

#### 3.2.5 The number of days when significant migration risk, forecast consultation or crop monitoring were advised

##### 3.2.5.1 a posteriori analysis

**Figure 2:**
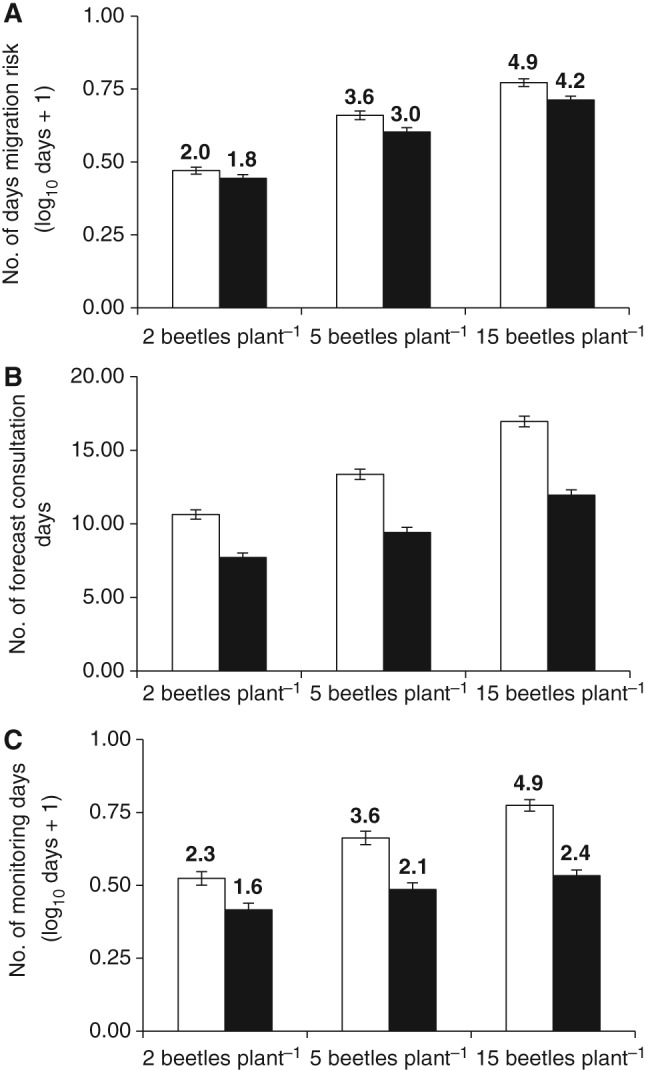
Comparison of advice for pollen beetle management, derived from rule‐based advice and from the DSS proPlant expert using recorded weather data a posteriori. (A) Number of days of migration risk advised up to breaches of different thresholds (log_10_ days + 1); (B) number of forecast consultation days advised up to the date that a threshold breach would be detected; (C) number of monitoring days advised up to the date that a threshold breach would be detected (log_10_ days + 1). Error bars = SED for each pair of means; numbers above bars are backtransformed means. Differences between each pair of means are significant (P < 0.001 in each case); for days of migration risk, F_2,39_ = 48.6, 112.2 and 182.4 for 2, 5 and 15 beetles plant^−1^, respectively; for consultation days, F_2,39_ = 77.0, 94.5 and 146.1, respectively; for monitoring days, F_2,39_ = 131.3, 148.8 and 187.8, respectively. 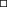 rule‐based advice; 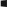 proPlant expert DSS.

The DSS proPlant expert consistently advised significantly fewer days of pollen beetle migration risk (yellow or red dots, ‘good’ or ‘optimum’ migration conditions, respectively) than did rule‐based advice (days with a temperature of ≥15 °C). At the 2, 5 and 15 beetles plant^−1^ thresholds, proPlant expert advised 9, 16 and 15% fewer risk days, respectively (Fig. 2A). Significantly fewer days of forecast consultation (for the DSS, consultation of proPlant expert's migration risk forecast; for rule‐based advice, consultation of the weather forecast) were advised by the DSS than by rule‐based advice (28–30% fewer) (Fig. 2B). The most marked difference in user input associated with the two management tools was a significant reduction in the number of days when crop monitoring was recommended by the DSS, which advised 31, 43 and 51% fewer days monitoring than suggested by rule‐based advice at the 2, 5 and 15 beetles plant^−1^ thresholds, respectively (Fig. 2C).

**Figure 3:**
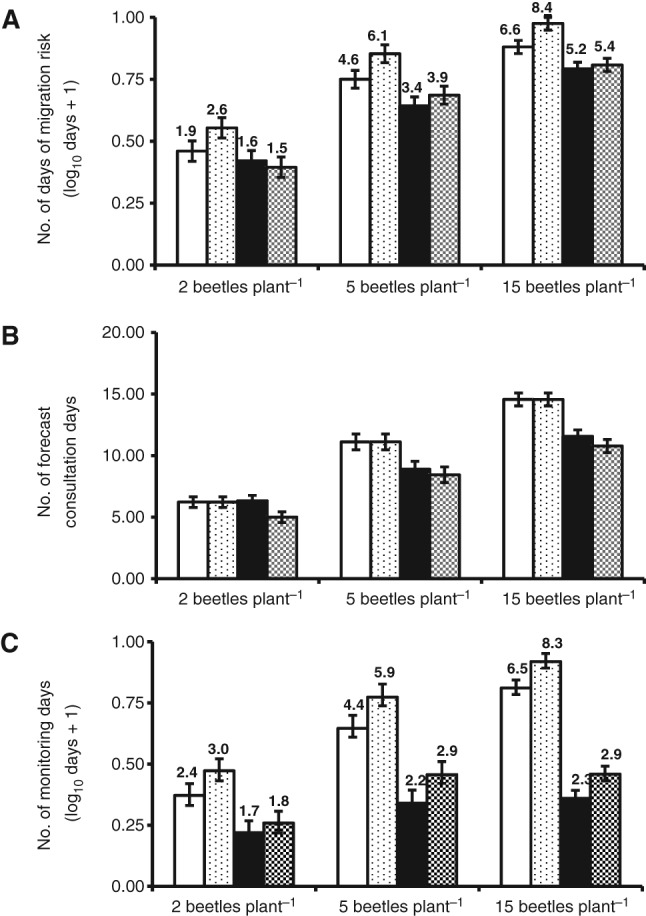
Comparison of advice for pollen beetle management derived from rule‐based advice and from the DSS proPlant expert using recorded weather data a posteriori or in real time. (A) Number of days of migration risk advised (log_10_ days + 1); (B) number of forecast consultation days advised; (C) number of monitoring days advised (log_10_ days + 1). Error bars = SED for each group of four means; numbers above bars are backtransformed means. Differences between each pair of means are significant; for days of migration risk F_2,24_ = 5.7, 12.9 and 19.8 with p = 0.004, < 0.001 and < 0.001 for 2, 5 and 15 beetles per plant, respectively; for consultation days, F_2,24_ = 4.3, 9.8 and 28.6, respectively with p = 0.015, < 0.001 and < 0.001; for monitoring days F_2,24_ = 11.3, 26.3 and 136.2, with p = < 0.001, < 0.001 and < 0.001, respectively. 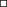 rule‐based advice a posteriori; 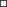 rule‐based advice real time; 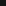 proPlant expert DSS a posteriori; 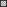 proPlant expert DSS real time.

*Real‐time analysi*s The comparison of real‐time advice and *a posteriori* advice at nine sites in the Bedford area in 2011 supported the results of the main analysis and validated the *a posteriori* approach. At almost every threshold level and both in real time and *a posteriori*, the DSS proPlant expert advised fewer days of good migration conditions, fewer days of forecast consultation and fewer pollen beetle monitoring days than associated with rule‐based advice (Fig. 3). Differences between the two systems in the real‐time analysis matched or exceeded those in the *a posteriori* analysis (Fig. 3).

## 4 DISCUSSION AND CONCLUSIONS

This study demonstrates that breaches of pollen beetle control thresholds can be reliably identified both using rule‐based advice and using the web‐based DSS proPlant expert. Moreover, by identifying a substantially reduced number of days for crop monitoring, proPlant expert could markedly reduce the potential labour cost of managing pollen beetle infestations according to control thresholds and encourage DSS uptake. This could facilitate the use of thresholds and lead to a reduction in unnecessary insecticide sprays.

Both rule‐based advice and the DSS proPlant expert performed well in prompting monitoring that would detect breaches of spray thresholds for pollen beetles in WOSR. However, at the 15 beetles plant^−1^ threshold, proPlant expert DSS advised 29% fewer forecast consultations than rule‐based advice, and 15% fewer risk days with good migration conditions. proPlant expert also specifically identified a subset of migration risk days on which it advised pollen beetle monitoring. These amounted to 51% fewer than the number of days with temperatures of ≥15 °C, the flight threshold temperature assumption underlying rule‐based advice. This is potentially of great significance for time‐pressured growers and crop consultants, for whom the labour cost associated with using threshold‐based advice is an important determinant of uptake when set against the low cost of many insecticides.^25^ In practice, it is unlikely that a user could or would monitor the crop every day with temperatures of ≥15 °C, which we have assumed to be necessary to implement rule‐based advice fully. However, the clear identification of a subset of optimal monitoring days by the proPlant expert DSS is likely to give the user confidence that less frequent crop monitoring can be effective and that it is practical to use a DSS. The reduced need for user input is achieved without loss of effectiveness in detecting breaches of threshold, and with an average delay in threshold breach detection of less than a day compared with rule‐based advice. It seems likely that this small delay would be accompanied with little additional risk to yield, given the strong compensatory ability of the crop,^26^, ^27^, ^28^ and that this would be outweighed by the benefit of using the DSS.

proPlant expert probably advises fewer days of migration risk because its model (based on 20 years of phenological data) takes into account more weather variables than the temperature‐based rule system we used in comparison. For example, a day might be warm enough for migration but too windy. The proPlant expert DSS also gives the user early warning of potential risk, indicating the earliest date that migration could start. In this study, proPlant expert's first migration risk warning consistently preceded or accompanied the first pollen beetles sampled on crops or on sticky traps. The date of the first proPlant expert dot preceded the first beetle caught by an average of 9.5 days in this study (supporting information Table S1). This could assist forward planning for growers and advisors, especially those using monitoring traps to determine local movement of pollen beetles or their abundance.

The phenological model underlying proPlant expert allows the DSS to provide not only warnings of good migration conditions but also estimates of the progress of migration on the specific days it recommends for monitoring. When combined with the numbers of pollen beetles observed in field monitoring, this may give the user confidence to target decision‐making and control activities more effectively. For example, if there are two beetles per plant in a crop and migration is predicted to be 90% complete, the risk of any new migration breaching, say, a 5 beetles plant^−1^ threshold must be less than if migration were only 40% complete. The user might decide that the need for control is so unlikely when migration is 90% complete that no further monitoring is needed.

Most comparisons of the two systems presented here were made *a posteriori*, using known weather data and known pollen beetle phenology. The validity of this approach was confirmed by the real‐time study, in which the reductions in the number of days of migration risk, forecast consultation and monitoring advised by the DSS compared with rule‐based advice matched or exceeded the reductions found *a posteriori*. Weather forecast data for both DSSs and for rule‐based advice should be chosen to reflect the conditions in the vicinity of the crop to which the advice is applied. proPlant expert aims to use a network of weather stations so that all crops are within 30 km of a station. Modern weather models enable modelling of forecast data at a grid scale finer than that of physical weather stations, enabling the interpolation of forecast data at a truly local scale. In this study, the average distance between weather stations and crops was 16 km. The two risk management tools performed well in spite of the inclusion of 11 sites more than 30 km from their weather stations, including one 80 km away.

As expected, for both risk management tools, the accuracy with which they forecast good migration conditions moderately declined, relative to an *a posteriori* assessment, as they predicted further into the future. However, they remained 85% or more accurate 2 days ahead. Modern weather forecasting models achieve high degrees of accuracy in predicting temperature, the basis of rule‐based advice on pollen beetle migration risk. At 45 sites across the United Kingdom in 2010–2012, 86.6% of maximum temperature forecasts were accurate to within ±2 °C on the second day of forecast (Met Office, http://www.metoffice.gov.uk/about‐us/who/accuracy/forecasts). The degree of difference between proPlant expert's predictive advice in real time and its *a posteriori* advice at nine sites in 2011 was therefore consistent with the likely degree of inaccuracy in the weather forecasts driving proPlant's model.

In spring 2012, Bayer CropScience ran a trial version of proPlant expert on their UK website as part of their stewardship of insecticides, with positive feedback from a small sample of users.^21^ Wide introduction of proPlant expert to UK farmers and advisers would help to increase the adoption of control thresholds into practice, encouraged by its labour efficiency and support for decision‐making. This should lead to better targeting of insecticides and reductions in insecticide use, as in Germany,^29^ and could make a significant contribution to managing insecticide resistance.^19^

A recent study on temperature–activity relationships in pollen beetles may provide scope to improve decision support models.^16^ Field studies have led to estimates of flight temperature thresholds in the range 10–15 °C, but are likely to be influenced by various meteorological and environmental variables and to be subject to the sampling effect.^12^, ^13^, ^14^, ^15^ Under controlled conditions in the laboratory, Ferguson *et al.* 16 showed that the relationship between temperature and flight followed a sigmoid curve over the range 6–23 °C rather than a simple threshold response. The lower half of this curve agreed well with the range of temperatures at which first flight has been reported in the field: 10% of beetles flying in the range 10.9–12.5 °C and 50% of beetles flying in the range 15.5–16.2 °C, similar to the 15 °C temperature at which rule‐based advice warns of significant migration risk.^9^, ^10^ These data could be used further to refine the accuracy of local‐weather‐based phenological models for pollen beetle that underpin web‐based DSSs.

Temperature also influences the severity of bud damage when pollen beetles arrive on the crop. Ferguson *et al.* 16 demonstrated strong positive relationships between temperature and the rates of feeding and oviposition on OSR buds. Such data offer scope to refine risk models to allow for the effect of weather on damage rates. The ability of the crop to compensate for pollen beetle damage is also influenced by temperature and other meteorological factors, as well as by growth stage^27^, ^30^, ^31^ and crop plant density,^28^ and these factors should also be taken into account. (Indeed, the most up‐to‐date advice available in the United Kingdom on risk assessment now builds plant density of the crop into risk assessment,^11^ based on preliminary evidence that pollen beetle risk is negatively related to plant density.^28^) Ultimately, a risk model driven not only by automatically downloaded local weather data and forecasts but also by plant growth stage could provide information on the severity of damage risk, as well as of migration risk, and might even offer a control threshold tailored to local crop and weather conditions. To underpin such developments, more work would be needed to quantify the effects of weather, growth stage and agronomic factors on winter OSR's ability to compensate for bud damage. In the long term, it may even be able to predict the abundance and distribution of pollen beetles by modelling their reproductive success the previous year, predation and overwintering mortality and their movement within the landscape.

For any DSS, reliability in offering accurate and effective timing of monitoring and control while minimising sampling effort and pesticide applications will remain key to engendering confidence among farmers and to encouraging them to adopt control thresholds more fully. A DSS based on a phenological model offers an approach to fulfil this role in pollen beetle management.

## Supporting information


**Figure S1.** Location of pollen beetle sampling sites used in this study.

**Table S1.** Comparison between migration indicators of the number of days elapsed between the date of first indication of migration and the first detection of a pollen beetle on traps or plants.

## References

[1] Heimbach U and Mueller A , Incidence of pyrethroid‐resistant oilseed rape pests in Germany. Pest Manag Sci 69(2):209–216 (2013). 23345124 10.1002/ps.3351

[2] Nauen R , Zimmer CT , Andrews M , Slater R , Bass C , Ekbom B *et al.*, Target‐site resistance to pyrethroids in European populations of pollen beetle, *Meligethes aeneus* F. Pestic Biochem Phys 103(3):173–180 (2012).

[3] Thieme T , Heimbach U and Muller A , Chemical control of insect pests and insecticide resistance in oilseed rape, in Biocontrol‐based Integrated Management of Oilseed Rape Pests, ed. by Williams IH Springer‐Verlag, London, UK, pp. 131–335 (2010).

[4] Zimmer CT and Nauen R , Pyrethroid resistance and thiacloprid baseline susceptibility of European populations of *Meligethes aeneus* (Coleoptera: Nitidulidae) collected in winter oilseed rape. Pest Manag Sci 67(5):599–608 (2011). 21394884 10.1002/ps.2137

[5] Alford DV , Nilsson C and Ulber B , Insect pests of oilseed rape crops, in Biocontrol of Oilseed Rape Pests, ed. by Alford DV Blackwell Science, Oxford, UK, pp. 9–42 (2003).

[6] Williams IH , The major insect pests of oilseed rape in Europe and their management: an overview, in Biocontrol‐based Integrated Management of Oilseed Rape Pests, ed. by Williams IH Springer‐Verlag, London, UK, pp. 1–43 (2010).

[7] Garthwaite DG , Thomas MR , Parrish G , Smith L , Chippindale C and Pietraville S , Pesticide Usage Survey Report 235 – Arable Crops in the United Kingdom 2010. Defra, London, UK (2011).

[8] Garthwaite DG , Thomas MR , Parrish G , Smith L and Barker I , Pesticide Usage Survey Report 224 – Arable Farm Crops 2008. Defra, London, UK (2009).

[9] Controlling pollen beetle and combating insecticide resistance in oilseed rape . HGCA Information Sheet 12, Spring 2011, Agriculture and Horticulture Development Board, Kenilworth, Warks, UK (2011).

[10] Oakley J , Pest management in cereals and oilseed rape – a guide (now archived). HGCA, Kenilworth, Warks, UK (2003).

[11] Monitoring and control of pollen beetle in oilseed rape . HGCA Information Sheet 18, Spring 2013, Agriculture and Horticulture Development Board, Kenilworth, Warks, UK (2013).

[12] Fritzsche R , Zur Biologie und Ökologie der Rapsschädlinge aus der Gattung *Meligethes* . Z Angew Entomol 40(2):222–280 (1957).

[13] Nilsson C , The pollen beetle (*Meligethes aeneus*) in winter and spring rape at Alnarp 1976–1978. I. Migration and sex ratio. Vaxtskyddsnotiser 52:134–139 (1988).

[14] Šedivy J and Kocourek F , Flight activity of winter rape pests. J Appl Entomol 117:400–407 (1994).

[15] Ferguson AW , Holdgate R , Mason NS , Clark SJ and Williams IH , Phenologies and dial periodicities of within‐crop flight by pests and parasitoids in winter oilseed rape in the UK. Bull IOBC/WPRS 92:45–54 (2013).

[16] Ferguson AW , Nevard LM , Clark SJ and Cook SM , Activity–temperature relationships in *Meligethes aeneus*: implications for pest management. Pest Manag Sci 71(3):459–466 (2014). 25052810 10.1002/ps.3860 PMC4345434

[17] Johnen A , Williams IH , Nilsson C , Klukowski Z , Luik A and Ulber B , The proPlant decision support system: phenological models for the major pests of oilseed rape and their key parasitoids in Europe, in Biocontrol‐based Integrated Management of Oilseed Rape Pests, ed. by Williams IH Springer‐Verlag, London, UK, pp. 381–403 (2010).

[18] Knight JD , The role of decision support systems in integrated crop protection. Agric Ecosyst Environ 64(2):157–163 (1997).

[19] Stratonovitch P , Elias J , Denholm I , Slater R and Semenov MA , An individual‐based model of the evolution of pesticide resistance in heterogeneous environments: control of *Meligethes aeneus* population in oilseed rape crops. PLoS ONE 9(12):e115631 (2014). 25531104 10.1371/journal.pone.0115631 PMC4274105

[20] Johnen A and von Richthofen J‐S , The decision‐support system proPlant expert: a computer‐based tool for integrated pest management in Europe. Bull IOBC/WPRS 96:99–105 (2013).

[21] Ferguson AW and Cook SM , Results of a small survey amongst farmers and advisers in the UK on their evaluation of the proPlant pollen beetle migration tool and its influence on their practice. Bull IOBC/WPRS 104:97–104 (2014).

[22] Richardson DM , Summary of findings from a participant country pollen beetle questionnaire. Bull EPPO 38:68–72 (2008).

[23] Cook SM , Doring TF , Ferguson AW , Martin JA , Skellern MP , Smart LE *et al.*, Developing an integrated pest management strategy for pollen beetles in winter oilseed rape – a UK Defra SA LINK project (LK09108) (HGCA RD‐2007‐3394). Bull IOBC/WPRS 96:45–45 (2013).

[24] Lancashire PD , Bleiholder H , Vandenboom T , Langeluddeke P , Stauss R , Weber E *et al.*, A uniform decimal code for growth‐stages of crops and weeds. Ann Appl Biol 119(3):561–601 (1991).

[25] Ellis S , Berry P and Walters K , A review of invertebrate pest thresholds. Research Review No. 73, HGCA, Kenilworth, Warks, UK (2009).

[26] Axelson J and Nielsen PS , Compensation in spring sown oilseed rape after attack by pollen beetles (*Meligethes aeneus* F.). Tidsskrift Planteavl 94(2):195–199 (1990).

[27] Williams IH and Free JB , Compensation of oilseed rape (*Brassica napus* L.) plants after damage to their buds and pods. J Agric Sci 92:53–59 (1979).

[28] Ellis S and Berry P , Re‐evaluating thresholds for pollen beetle in oilseed rape. HGCA Publication PR495, AHDB‐HGCA, Kenilworth, Warks, UK (2012).

[29] Johnen A , Williams IH , Ferguson AW , Büchs W , Klukowski Z , Nilsson C *et al.*, MASTER: validation of existing phonological models of the proPlant DSS for key pests in winter oilseed rape in different climatic areas of Europe and prospects for IPM. Proc Int Symp – Integrated Pest Management in Oilseed Rape, 3–5 April 2006, Göttingen, Germany, pp. 140–142 (2006).

[30] Nilsson C , Yield losses in summer rape caused by pollen beetles (*Meligethes* spp.). *Swed* J Agric Res 17(3):105–111 (1987).

[31] Hansen LM , Economic damage threshold model for pollen beetles (*Meligethes aeneus* F.) in spring oilseed rape (*Brassica napus* L.) crops. Crop Prot 23(1):43–46 (2004).

