# Supplementary material for: The potential of decision support systems to improve risk assessment for pollen beetle management in winter oilseed rape
Source: Pest Manag Sci. 2015 Aug 26;72(3):609–17. doi: 10.1002/ps.4069 (PMC5049606; doi:10.1002/ps.4069)
Supplement: Supplementary file 2 — Table S1. Comparison between migration indicators of the number of days elapsed between the date of first indication of migration and the first detection of a pollen beetle on traps or plants. [file PS-72-609-s002.docx]

**SUPPORTING INFORMATION**

Table S1. Comparison between migration indicators of the number of days elapsed between the date of first indication of migration and the first detection of a pollen beetle on traps or plants.

|  | ≥15 °C first recorded | Date of first proPlant dot of any colour | Date of first proPlant yellow or red dot |
| --- | --- | --- | --- |
| Mean days | 0.20 | 9.50 | -2.77 |
| SEM | 4.669 | 3.678 | 4.986 |
| n (number of fields) | 44 | 44 | 44 |
| % fields where  indicator warned on or before date of first migration | 61.4 | 100.0 | 50.0 |
